# Traditional fermented foods of Indonesia harbour functionally redundant but phylogenetically diverse taxa

**DOI:** 10.1093/femsmc/xtag005

**Published:** 2026-01-22

**Authors:** Wisnu Adi Wicaksono, Elma Zukancic, Matevz Zlatnar, Antonius Suwanto, Gabriele Berg

**Affiliations:** Institute of Environmental Biotechnology, Graz University of Technology, Graz 8010, Austria; Institute of Environmental Biotechnology, Graz University of Technology, Graz 8010, Austria; Institute of Environmental Biotechnology, Graz University of Technology, Graz 8010, Austria; Department of Biology, Faculty of Mathematics and Natural Science, IPB University, Bogor 16680, Indonesia; Institute of Environmental Biotechnology, Graz University of Technology, Graz 8010, Austria; Leibniz Institute for Agricultural Engineering and Bioeconomy (ATB), Potsdam 14469, Germany; Institute for Biochemistry and Biology, University of Potsdam, Potsdam 14476, Germany; Department of Colloid Chemistry, Max Planck Institute of Colloids and Interfaces, Potsdam 14476, Germany

**Keywords:** fermented food, rice, cassava, shrimp, microbiome, metagenome, metabolic model

## Abstract

Fermented foods represent complex microbial ecosystems that contribute to food quality, functionality, and potential health benefits, yet many traditional fermented foods remain poorly characterized. The aim of this study was to study microbial diversity, and functional potential of underexplored traditional Indonesian fermented food. The fermented products displayed substantial variation in bacterial richness, ranging from 65 to 614 bacterial amplicon sequence variants across samples. The microbial communities were dominated by bacterial taxa affiliated with the orders *Bacillales* and *Lactobacillales*, alongside fungal taxa from the order *Mucorales*. The plant-based products i.e. tape ketan and tape singkong had a higher bacterial abundance but lower diversity than animal-based terasi. We found significant correlations between bacterial and fungal communities dominated by positive cooccurrence patterns and highly complex networks especially in terasi. Each food product was characterized by a unique functional profile of genes linked to beneficial metabolic functions (biosynthesis of bacteriocins, short-chain fatty acids, and vitamins) but tape ketan samples demonstrated the highest diversity and abundance of them. Metagenome assembled genomes reflect a high diversity of health beneficial properties as well as substrate-specific degradation capabilities. Traditional Indonesian fermented foods harbour functionally redundant but phylogenetically diverse taxa offering a potential source for probiotic traits and functional food development.

## Teaser

This study investigates the microbial and functional diversity of traditional fermented foods in Indonesia. Plant-based fermented products demonstrated higher bacterial abundance, but lower diversity compared to animal-based terasi.

## Introduction

Food fermentation has been an integral part of human cultures. This fermentation process, driven by the activity of bacteria and fungi, enhances the flavour and texture of the final products while also improving their nutritional value. In recent years, scientific research has increasingly focused on the health benefits of fermented foods, particularly due to their probiotic content and bioactive compounds, such as bacteriocin, short-chain fatty acids (SCFAs), and vitamins (Marco et al. 2017). For example, regular kimchi consumption improved lipid profiles, increased adiponectin levels, and reduced inflammation-related markers. It also increased the abundance of SCFA–producing genera (*Faecalibacterium, Roseburia*, and *Phascolactobacterium*) while reduced the abundance of *Clostridium* spp. and *Escherichia coli* (Kim and Park 2018). Consumption of yogurt significantly increased *Lactobacillus* and *Bifidobacteria* levels after 14 days (Liao et al. 2022). Additionally, acetic acid, total SCFAs, and SIgA levels were higher in individuals with functional constipation and diarrhea, indicating improved intestinal immune function. These findings underscore the potential of fermented foods as functional ingredients for supporting metabolic and gut health and preventing diseases associated with the disappearing microbiota in the human gut (Blaser and Dominguez-Bello 2025). Although interest in fermented foods is increasing, there is still a considerable knowledge gap regarding the microbial diversity, health benefits, and functional properties of traditional fermented foods.

In many regions across Asia, fermented foods have been integral to traditional dietary practices and food preservation methods. Unlike Western fermentation, which commonly focuses on bacterial cultures, numerous iconic Asian fermented products primarily depend on filamentous fungi such as *Aspergillus, Rhizopus, Mucor*, and *Monascus* for their enzymatic activities and flavour development. For example, in Japan and China, koji, a fermentation starter made with *Aspergillus oryzae* cultured on soybeans, is essential for producing soy sauce, miso, and sake (reviewed in Tamang et al. 2016). In Indonesia, tempeh, a fermented soybean product, is produced using *Rhizopus oligosporus*, which transform the soybeans into a firm, protein-rich mass that enhances digestibility and nutritional value (Nout and Kiers 2005). Despite the wide variety of fungi-based traditional fermented foods throughout Asia, scientific research has largely concentrated on soybean- and cabbage-based fermented products, resulting in limited characterization and documentation of many other traditional fermented foods within the international literature (Surya 2024). Moreover, recent studies have demonstrated that, although filamentous fungi serve as primary starter cultures in fermentation, bacterial communities also play significant ecological roles. For example, in tempeh, the associated bacterial populations exhibit distinct compositions influenced by their geographical origin (Wicaksono et al. 2024a). Similar patterns have been observed in koji fermentation, where variations in bacterial community structure are attributed to differences in environmental conditions, geographic location, and production methods (Zhao et al. 2022). These bacterial assemblages do not only contribute to acidification and inhibition of spoilage organisms but also to the synthesis of bioactive metabolites, vitamins, and enzymes (Wicaksono et al. 2024b, Leech et al. 2020, Liu et al. 2023) thereby enhancing the nutritional and sensory qualities of the final product. Deciphering the complex interactions between fungal starter cultures and their cooccurring bacterial communities will advance our understanding of traditional fermentation processes and offer promising opportunities to optimize the nutritional composition and probiotic potential of underexplored traditional fermented foods.

Traditional Indonesian fermented foods, such as tape ketan, tape singkong, and terasi have received comparatively little attention, particularly using integrated multiomics approaches. While there are some microbiological and culture–based studies on tape ketan and tape singkong that document lactic acid bacterial community and sensory properties (Hasanah et al. 2018), these studies are largely limited in scope and do not comprehensively characterize community structure and function. Similarly, although terasi has been characterized in terms of its fermentation process, dominant bacterial taxa, and biochemical changes, most studies have relied on culture–dependent methods (Herlina and Setiarto 2024), which likely underestimate the full microbial diversity and fail to capture the functional potential of the microbial community. This fragmented literature limits our understanding of the functional interactions among microbial taxa and their metabolic contributions during fermentation. Addressing this gap by applying a multiomics framework is therefore essential to elucidate complex bacterial–fungal interactions, functional potential, and metabolic outputs in these underexplored traditional fermented foods.

This study aimed to explore microbial diversity and functional potential, thereby providing new insights into the microbial communities of culturally significant, yet underexplored, traditional Indonesian fermented foods. In Indonesia, the diversity of traditional fermented foods in each production region reflects the significance of local agricultural products and cultural practices unique to that area. For example, tape ketan (fermented glutinous rice) and tape singkong (fermented cassava) are widely produced and consumed in Java, while terasi (fermented shrimp paste) is commonly produced and used in both Java and Sumatra. These fermented products constitute essential components of regional food systems and cultural heritage, contributing not only to food preservation but also to improved nutritional status (Surono 2016). Additionally, they function as condiments or appetizers and support the informal economic sector. Tape ketan and tape singkong fermentations are primarily driven by filamentous fungi such as *Amylomyces rouxii* and *Rhizopus* spp., which facilitate the conversion of complex carbohydrates into more digestible forms and generate bioactive compounds (Cempaka 2021). Terasi fermentation relies on complex microbial consortia, including halophilic bacteria and fungi (Herlina and Setiarto 2024), which contribute to its characteristic umami flavour and preservation qualities. This study employs an integrated multiomics approach combining quantitative polymerase chain reaction (qPCR), amplicon sequencing, shotgun metagenomics, and microbial community modelling to dissect the complex microbial community structures and interactions within tape ketan, tape singkong, and terasi.

## Materials and methods

### Experimental design, sample processing, and DNA extraction

Fermented food samples, i.e. tape ketan, tape singkong, and terasi (*n* = 44; Table S1) were collected from 15 traditional markets and supermarkets across six different regions, including West Java, DKI Jakarta, East Java, Central Java, West Nusa Tenggara, and Bali. For tape ketan samples, they were made from either black glutinous rice or white glutinous rice. This sampling strategy was designed to capture variation in different fermented foods (plant- versus animal-based). Detailed fermentation conditions were not recorded, as these products are prepared using diverse artisanal practices. Collecting samples from multiple regions and markets provides a broad overview of the microbial diversity in these foods. Each sample was subsequently homogenized manually with a sterile spatula to ensure uniform distribution of microbial content.

Approximately 50 g of each sample were aseptically collected using sterile forceps and transferred into DNase/RNase-free 50 ml polypropylene tubes (Falcon™, Corning, NY, USA). Immediately following homogenization, DNA/RNA Shield™ (Zymo Research, Freiburg, Germany) was added to each sample to maintain nucleic acid integrity. The tubes were vortexed for 10–15 s to ensure thorough immersion of the samples in the preservation buffer. Subsequently, the samples were stored at –20°C until further processing. All samples were subjected to DNA extraction after 10 days of storage under the specified conditions. Samples were categorized based on the type of raw material: glutinous rice (tape ketan), cassava (tape singkong), and planktonic shrimp (terasi). Additionally, the first two samples are plant-based fermented foods, while the latter is an animal-based fermented product. The homogenized samples were transferred to Lysing Matrix B tubes containing 0.1 mm diameter beads (MP Biomedicals, Irvine, CA, USA) and subjected to mechanical disruption for 30 s using a FastPrep- homogenizer (MP Biomedicals). Total DNA was extracted using the FastDNA SPIN Kit for Soil, following the manufacturer’s instructions. The extracted DNA was stored at −20°C until PCR analyses.

### Microbial quantification using qPCR

Quantitative real-time PCR (qPCR) utilizing SYBR Green fluorescence was employed to quantify microbial load in the samples, expressed as gene copies per gram. The primer sets used included 515f-806r (Caporaso et al. 2011) for total bacteria, ITS1f-ITS2r (White et al. 1990) for total fungi, and F-lac/R-lac (Walter et al. 2001) for lactobacilli. The reactions were prepared in a final volume of 10 µl, consisting of 5 μl of 2X KAPA SYBR Green (Kapa Biosystems, USA), 0.5 μl of each primer, 3 μl of PCR-grade water, and 1 μl of template DNA (diluted 1:10 in PCR-grade water). Quantitative PCR was carried out using the qTOWER 3 G (Analytik Jena GmbH, Jena, Germany). The thermal cycling conditions included an initial denaturation at 95°C for 10 min for lactobacilli, and 3 min for 16S rRNA and ITS. This was followed by 40 cycles consisting of denaturation at 95°C for 5 s, annealing at specific temperatures—60°C for lactobacilli, 54°C for 16S, and 58°C for ITS, extension at 72°C for 15 s, and a final melting curve. The calculated PCR efficiencies were 0.82% (*R*² = 0.993) for the 515f–806r primers, 0.85% (*R*² = 0.998) for the ITS1f–ITS2r primers, and 1.06% (*R*² = 0.997) for the F-lac/R-lac primers.

### Microbial community profiling using 16S rRNA and ITS amplicon sequencing

To profile the microbial community composition, we conducted 16S rRNA gene amplicon sequencing for bacteria and ITS region amplicon sequencing for fungi. The V4 hypervariable regions of the 16S rRNA gene and the ITS1 region were amplified from total DNA using the primer pair 515f-806r (Caporaso et al. 2011) for total bacteria and ITS1f-ITS2r (White et al. 1990) for total fungi, respectively. Each primer set included Illumina-compatible index sequences to facilitate multiplexing. Two technical replicates were conducted for each sample. The PCR reactions were carried out using a Mastercycler Nexus X2 PCR Thermocycler (Eppendorf), with specific cycling parameters for each target region as described below.

For the bacterial 16S rRNA gene amplification, the cycling conditions were as follows: an initial denaturation at 96°C for 5 min, followed by 35 cycles consisting of denaturation at 96°C for 60 s, annealing at 54°C for 60 s, and extension at 72°C for 60 s. For the ITS1 region amplification, the cycling conditions included an initial denaturation at 94°C for 3 min, followed by 30 cycles of denaturation at 94°C for 30 s, annealing at 52°C for 30 s, and extension at 68°C for 30 s. The PCR products were visualized on a 1% agarose gel to confirm successful amplification. These products were then pooled in equal molar concentrations and purified using the Wizard SV Gel and PCR Clean-Up System (Promega). The pooled PCR products were submitted to Novogene (Cambridge, UK) for library preparation. Sequencing was performed on an Illumina NovaSeq 6000 platform, generating 2 × 250 bp paired-end reads.

### Microbial functional profiling using shotgun metagenomic sequencing

To investigate the functional potential of microbial taxa present in the fermented food samples, we performed shotgun metagenomic sequencing on four representative samples, each corresponding to one of the fermented food types analysed. This included two variants of tape ketan that prepared from black glutinous rice and white glutinous rice. The extracted DNA was sent to Novogene, a sequencing service provider based in Germany. Novogene conducted the library preparation and performed sequencing using the Illumina NovaSeq 6000 platform with 2 × 150 bp paired-end reads.

### Bioinformatics analysis

For amplicon sequencing data, demultiplexing and primer removal were performed using Cutadapt (Martin 2011). Sequence quality filtering, denoising, and chimera removal were conducted in QIIME2 (Bolyen et al. 2019), employing the DADA2 plugin (Callahan et al. 2016). The resulting amplicon sequence variants (ASVs) were classified taxonomically using VSEARCH (Rognes et al. 2016) against the SILVA v138 (Quast et al. 2012) and UNITE V 8.3 (Abarenkov et al. 2010) databases for bacterial and fungal datasets, respectively. ASV tables and taxonomic assignments were used as inputs for community analyses. Sequences identified as chloroplast, mitochondrial, and nontarget taxa were excluded.

For shotgun metagenome sequencing data, before examining microbial functional diversity in fermented food samples, raw shotgun metagenomic reads underwent quality filtering using Trimmomatic v0.39 (Bolger et al. 2014). High-quality reads were used as input for microbial functional profiling with HUMAnN3 v3.1.1 (Beghini et al. 2021). Metagenome-assembled genomes (MAGs) were reconstructed using MaxBin2 v2.2.7, MetaBAT2 v2.12.1, and CONCOCT v1.1.0 (Alneberg et al. 2014, Wu et al. 2016, Kang et al. 2019), with integration performed via DASTool v1.1.1 (Sieber et al. 2018). The quality of MAGs was evaluated using CheckM v1.0.13 (Parks et al. 2015), retaining only medium-quality MAGs (≥50% completeness and ≤10% contamination) aligned with MIMAG standards (Bowers et al. 2017). Dereplication was employed using dRep v2.2.3 (Olm et al. 2017) to establish a nonredundant MAG collection. Taxonomic classifications were assigned using GTDB-Tk (Chaumeil et al. 2020), and phylogenetic relationships were inferred with PhyloPhlAn. Gene annotation was performed using METABOLIC (Zhou et al. 2022), and MAG abundance estimates were calculated with CoverM v0.4.0 (https://github.com/wwood/CoverM) using the RPKM (reads per kilobase per million reads) metric.

Genome-scale metabolic models of bacterial taxa were reconstructed using the automated tool CarveMe (Machado et al. 2018), with gap-filling applied to ensure all models are capable of growth in Luria–Bertani (LB) medium. For each fermented food product, community-level metabolic models were developed based on the five MAGs exhibiting the highest relative abundance, as determined from metagenomic sequencing data. To assess potential metabolic interactions and cross-feeding within each microbial community, simulations were performed using MICOM v0.37.1 (Diener et al. 2020). Growth simulations were conducted under defined media conditions specific to each fermented food product, incorporating nutritional data from the CIQUAL food composition database (https://ciqual.anses.fr). In particular, the media compositions were based on ‘rice, cooked, unsalted’ for tape ketan, ‘cassava or manioc roots, cooked’ for tape singkong, and ‘shrimp or prawn, raw’ for terasi, each supplemented with LB medium to support microbial growth. Flux balance analysis was performed within the MICOM framework using the fluxes function to simulate microbial growth dynamics and to predict potential metabolic exchanges among community members.

### Statistical analysis

Statistical analyses and visualizations were performed in R version 4.4.3 (Core Team 2013) using pyhloseq package (McMurdie and Holmes 2013). Differences in bacterial gene copy numbers, richness, and diversity between groups were assessed using the Kruskal–Wallis test (*P* < .05). Bacterial richness was determined by calculating the number of ASVs (*n*_ASV_), and bacterial diversity was calculated using the Shannon diversity index (H). Multiple comparisons were conducted with Dunn’s test, with *P*-values adjusted using the false discovery rate correction. To assess bacterial alpha diversity, the dataset was normalized by randomly subsampling to the lowest read count across all samples. For beta diversity analysis, the dataset was normalized using MetagenomeSeq’s cumulative sum scaling (CSS; Paulson et al. 2013), and Bray–Curtis dissimilarity matrices were constructed from the CSS normalized data. These dissimilarity matrices were subsequently analysed using Permutational ANOVA (PERMANOVA) via the Adonis function to assess the impact of experimental factors on bacterial community composition. Further, a Procrustes analysis was conducted using the ‘protest’ function in the vegan package (Oksanen et al. 2007) to assess the concordance between fungal and bacterial communities. Concordance reflects the similarity in β-diversity between the two communities across samples in the same fermented food products.

To explore potential interactions between bacterial and fungal taxa, we performed a cooccurrence network analysis based on genus-level relative abundance data. ASV tables for bacterial and fungal communities were first aggregated to the genus level and then merged into a single compositional abundance matrix. Low-abundance genera (present in fewer than 25% of samples or with mean relative abundance < 0.01%) were filtered out to reduce noise. The SPIEC-EASI analysis (Kurtz et al. 2015) was employed to infers correlations between microbial taxa from compositional data. The resulting network was visualized using the igraph package (Csardi and Nepusz 2006). Network topology, including metrics such as degree centrality, clustering coefficient, and modularity, was further analysed using NetworkAnalyzer in Cytoscape 3.8.2 (Saito et al. 2012).

## Results

### Microbial abundance and diversity are influenced by the type of fermented product

**Figure 1:**
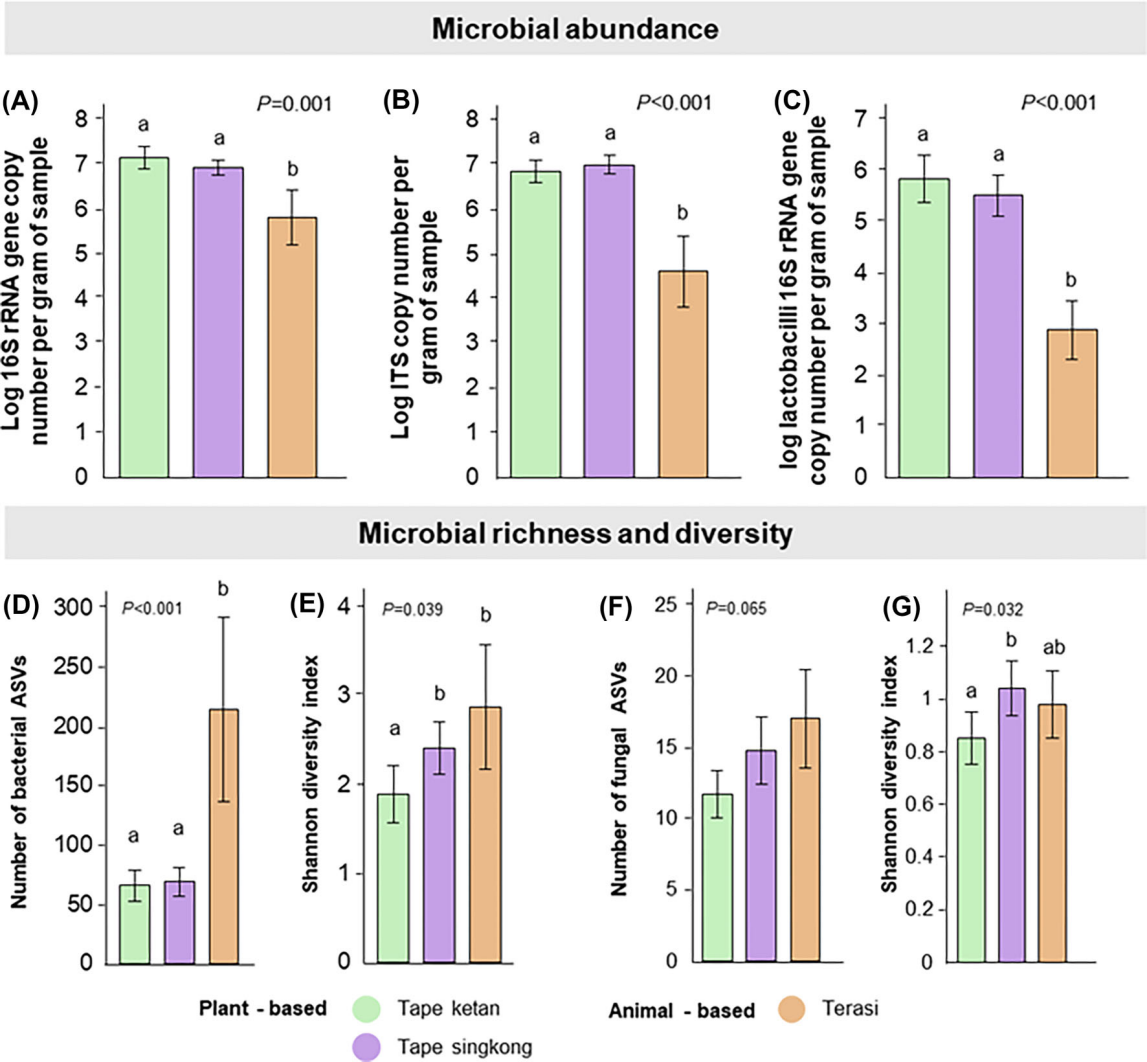
Comparison of microbial abundance, richness and diversity in different fermented food products. Quantitative PCR was employed to assess total bacterial load (A), fungi (B), and lactobacilli (C). High-throughput amplicon sequencing was used to determine the microbial richness (D—bacterial richness and F—fungal richness) and diversity (E—bacterial Shannon diversity index and G—fungal Shannon diversity index).

Plant-based fermented products, i.e. tape ketan and tape singkong, had a higher microbial abundance but lower microbial richness compared to terasi (Fig. 1). Specifically, tape ketan (*P*_adjusted_ = 0.002; mean 1.4 × 10^7^ 16S rRNA gene copies g⁻¹) and tape singkong (*P*_adjusted_ = 0.002; mean 8.3 × 10^7^ 16S rRNA gene copies g⁻¹) demonstrated significantly higher total bacterial abundance, compared to terasi (6.5 × 10^5^ 16S rRNA gene copies g⁻¹; Fig. 1A). A similar trend was also observed for fungal abundance from these products. Tape ketan (*P*_adjusted_ < 0.001; mean 7.1 × 10^6^ ITS gene copies g⁻¹) and tape singkong (*P*_adjusted_ < 0.001; mean 9.8 × 10^6^ ITS gene copies g⁻¹) demonstrated significantly higher total bacterial abundance, compared to terasi (3.9 × 10^4^ ITS copies g⁻¹; Fig. 1B). On average, lactobacilli abundance was also substantially lower in terasi (7.6 × 10^2^ 16S rRNA gene copies g⁻¹; Fig. 1C) compared to tape ketan (*P*_adjusted_ < 0.001; 6.9 × 10^5^ 16S rRNA gene copies g⁻¹) and tape singkong (*P*_adjusted_ < 0.001; and 3.3 × 10^5^ 16S rRNA gene copies g⁻¹). Interestingly, comparative analyses revealed contrasting patterns of bacterial richness and diversity among the samples (Fig. 1D and E). Terasi samples exhibited significantly higher bacterial richness (*n*_ASV_ = 214) than both tape ketan (*P*_adjusted_ < 0.001; *n*_ASV_ = 65.8) and tape singkong (*P*_adjusted_ < 0.001; *n*_ASV_ = 68.9). Moreover, terasi samples also demonstrated a significantly (*P*_adjusted_ = 0.049) higher bacterial diversity (H = 2.9) compared to tape ketan (H = 1.9). Although terasi samples also tended to have higher fungal richness and diversity compared to tape ketan, the differences were not statistically significant (Fig. 1F and G; Kruskal–Wallis test—*P* > .05). We observed a significantly higher fungal diversity in tape singkong compared to tape ketan (*P*_adjusted_ = 0.038).

### Microbial community composition varies by fermented food type

**Figure 2:**
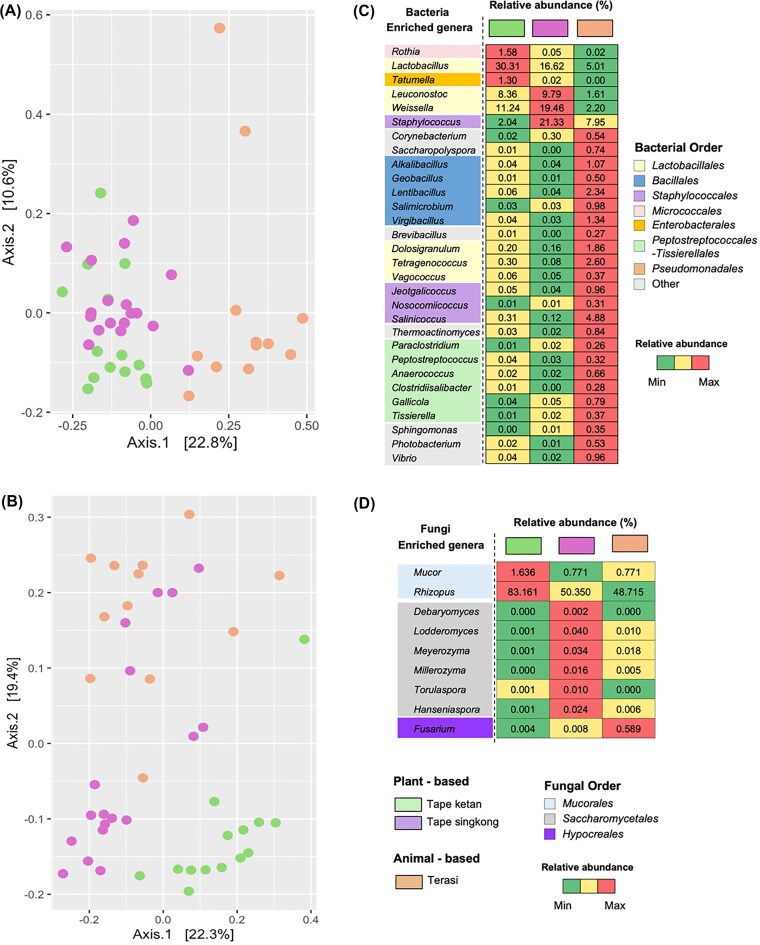
Microbial community clustering and differential abundance analysis of taxa across various fermented foods. The PCoA plots illustrate the clustering of bacterial (A) and fungal (B) communities from the fermented foods. Linear discriminant analysis effect size (LEfSe) was utilized to determine bacterial (C) and fungal (D) biomarkers at the genus level for each fermented food.

The type of fermented food influenced the composition of microbial communities. The Adonis analysis revealed that the type of fermented product accounted for 22.8% and 24.8% of the variation observed in bacterial and fungal communities, respectively. Supporting these results, the PCoA plots showed clear clustering based on the types of fermented foods (Fig. 2A and B). Plant-based fermented foods, such as tape ketan and tape singkong, tended to cluster together, whereas the animal-based fermented product, terasi, formed a distinct cluster. Nonetheless, pairwise Adonis analysis revealed that tape singkong and tape ketan possess significantly different bacterial and fungal community structures *P*_adjusted_ = 0.003).

The bacterial communities in the fermented foods were predominantly composed of members from the orders *Bacillales* and *Lactobacillales* (Fig. S1A). The fungal communities were primarily characterized by the orders *Mucorales*, including the genus *Rhizopus*, and *Saccharomycetales*, including the genus *Saccharomycopsis*. Tape ketan and tape singkong had high relative abundances of *Lactobacillales* (67.3% and 59.1%, respectively), whereas terasi was dominated by *Bacillales* (49.2%). *Staphylococcales* was also present in high abundance in tape singkong (21.5%) and terasi (13.4%). The fungal compositions of tape singkong and terasi were notably similar, primarily dominated by the genus *Rhizopus*, with mean relative abundances of 50.3% and 48.7%, respectively (Fig. S1B). Additionally, *Saccharomycopsis* was present at comparable levels. In contrast, tape ketan exhibited a distinct microbial profile, with *Rhizopus* being the most prevalent genus at ~83.1%.

**Figure 3:**
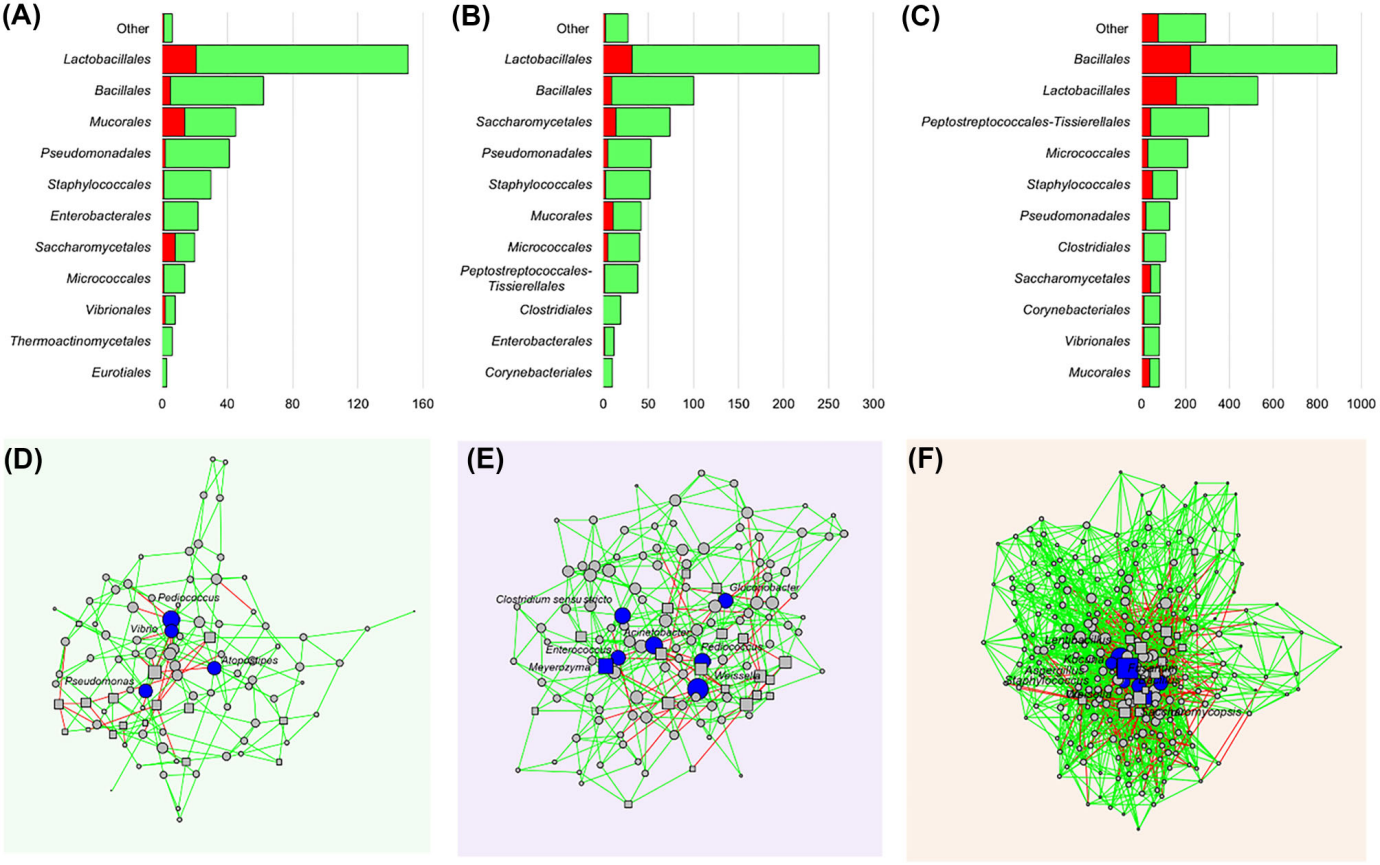
Network analysis of microbial communities. List of taxa with positive and negative interaction and cooccurrence network analysis from tape ketan (A), tape singkong (B), and terasi (C). Network analysis of microbial communities from tape ketan (D), tape singkong (E), and terasi (F) samples. A connection indicates a statistically significant correlation (*P* < .05) between two ASVs. Red lines denote negative correlations, while green lines indicate positive correlations. Different shapes represent distinct microbial domains: circles for bacteria and squares for fungi. Blue nodes identify key microbial taxa acting as hubs within the network.

Differential abundance analysis revealed that specific taxa are more closely associated with certain fermented foods. For example, the well-known lactic acid bacteria (LAB) taxon *Lactobacillus* was present at significantly higher levels in tape ketan compared to tape singkong and terasi (Fig. 2C). In contrast, other LAB taxa such as *Leuconostoc* and *Weissella* were more prevalent in tape singkong. Terasi sample was predominantly characterized by genera within the order *Bacillales*, including *Alkalibacillus, Virgibacillus*, and *Lentibacillus* (Fig. 3A). Additionally, various *Lactobacillales* taxa such as *Dolosigranulum, Tetragenococcus*, and *Vagococcus* were detected at substantially higher levels in terasi compared to plant-based fermented foods. Notably, halophilic Gram-positive bacteria, such as *Salinicoccus* and *Jeotgalicoccus*, were also more abundant in terasi relative to plant-based fermented products. Specific taxa belonging to certain fungal orders were associated with different fermented foods (Fig. 2D). Two fungal genera within the order *Mucorales* namely *Rhizopus* and *Mucor*, were found to be more prevalent in tape ketan. Notably, several fungal taxa within the class *Saccharomycetales*, including *Lodderomyces, Meyerozyma*, and *Hanseniaspora*, were more abundant in tape singkong. In contrast, *Fusarium*, which belongs to the order *Hypocreales*, was more abundant in terasi compared to plant-based products.

### Significant bacterial–fungal interactions were observed in tape singkong and terasi

Our analysis indicated a significant correlation between bacterial and fungal community compositions in certain fermented food samples. The Procrustes analysis revealed that bacterial and fungal communities exhibited significantly similar spatial structure in tape singkong (*M*^2^ = 0.625, *r* = 0.612, *P* = .001) and terasi (*M*^2^ = 0.642, *r* = 0.598, *P* = .029). However, the same pattern was not observed for tape ketan samples (*M*^2^ = 0.950, *r* = 0.223, *P* = .692).

We then aimed to evaluate bacterial and fungal interactions by constructing microbial cooccurrence networks. The microbial cooccurrence network for all fermented foods predominantly exhibited positive correlations (Fig. 3A–C). For instance, the microbial cooccurrence network derived from tape ketan had 176 positive and 28 negative correlations whereas had the microbial cooccurrence network derived from terasi had 1205 positive and 368 negative correlations. Interestingly, the microbial cooccurrence network derived from tape singkong had the highest ratio of positive negative correlation (7.6–312 positive and 41 negative correlations). We also observed a higher ratio of positive negative correlation between bacterial and fungal ASVs in tape singkong (4.66) compared to tape ketan (2.58) and terasi (1.14). In the network analysis of tape ketan samples, two predominant bacterial orders, *Lactobacillales* (*Bacilli*) and *Bacillales* (*Bacilli*), exhibited a higher number of connections, with 151 and 62 edges respectively, compared to other taxa. These same taxa also had the highest number of connections in the networks derived from tape singkong samples (*Lactobacillales* with 239 edges and *Bacillales* with 100 edges) and terasi samples (*Lactobacillales* with 529 edges and *Bacillales* with 890 edges).

Based on topological parameters, the networks derived from tape ketan and tape singkong samples, with 95 and 126 nodes, respectively, were less complex (clustering coefficient 0.170 and 0.144, respectively; Fig. 3D and E). In contrast, the microbial network derived from terasi samples exhibited greater complexity and density, characterized by the highest number of nodes (259) and edges (1573; Fig. 3F). The network also exhibited a high clustering coefficient (0.198). Interestingly, network derived from terasi displayed a higher heterogeneity (0.420) and centralization (0.101) compared to tape ketan (heterogeneity = 0.344, centralization = 0.062) and tape singkong (heterogeneity = 0.321, centralization = 0.060). This result indicated that microbial community in terasi was organized around a highly connected and central taxa (Fig. 3F).

Bacterial taxa exhibited strong cooccurrence patterns, forming a well-connected microbial network. The microbial network derived from terasi samples was dominated by dominant bacterial ASVs identified as *Lentibacillus* and *Staphylococcus* (mean relative abundances: 9.2% and 3.8%, respectively; Fig. 3F). Interestingly, low abundant bacterial ASVs that classified as *Kocuria* (mean relative abundances: 0.03%) and *Bacillus* (mean relative abundances: 0.2%) were identified as key hubs indicating their potential ecological importance despite low representation. Fungal ASVs, including *Fusarium* (mean relative abundances: 0.3%) and *Aspergillus* (mean relative abundances: 0.07%), also emerged as key hubs within the network derived from terasi samples (Fig. 3F). In the network derived from tape ketan samples, a hub ASV assigned to *Pediococcus* was identified, which was also predominant in the community (mean relative abundances: 10.2%). Other low-abundance bacterial ASVs such as *Vibrio* (mean relative abundances: 0.02%), *Atopostipes* (mean relative abundances: 0.05%), and *Pseudomonas* (mean relative abundances: 0.01%) were also characterized as hub taxa, suggesting their potential functional relevance. In tape singkong samples, dominant ASVs identified as *Pediococcus* and *Weissella* were identified as key hub taxa. Notably, ASVs classified as potentially opportunistic pathogenic genera namely *Clostridium* and *Acinetobacter* were detected as network hubs highlighting the complex ecological roles that opportunistic microbes may play within the fermentation ecosystem.

### Distinct functional profiles and enrichment of health-related genes in fermented food microbiome

**Figure 4:**
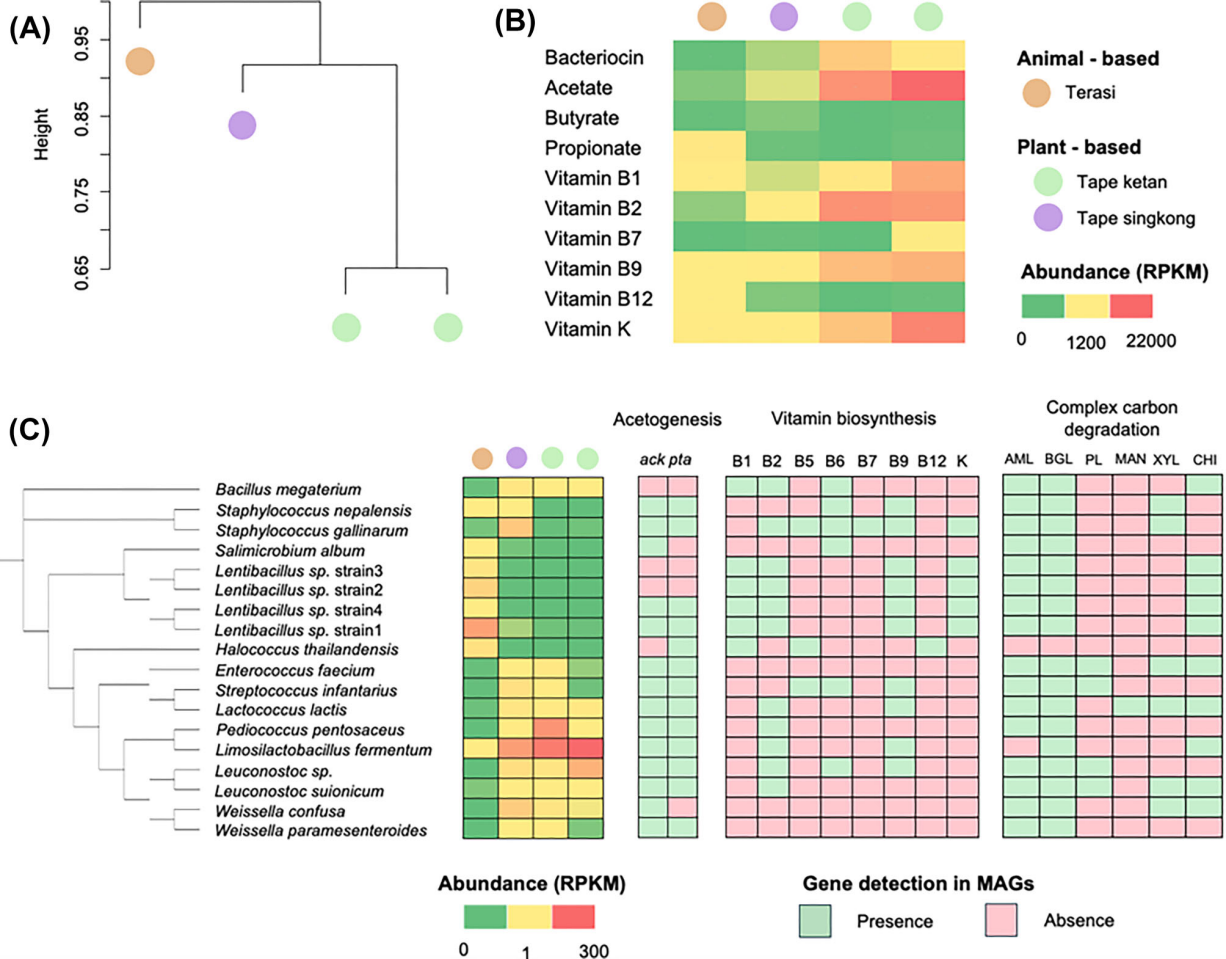
Hierarchical clustering of functional profiles, gene abundances associated with beneficial functions, and MAG profiles. Beta diversity was evaluated using a Bray–Curtis dissimilarity matrix based on gene functional profiles and was visualized through hierarchical clustering analysis (A). Selected gene functional profiles associated with beneficial functions across various fermented foods (B). A phylogenetic tree was developed to illustrate the diversity of the recovered MAGs and the gene profiles associated with selected functions in MAGs derived from the fermented food metagenome (C). The presence or absence of specific genes was visualized using colour-coded plots, highlighting the gene profiles within the MAGs obtained from the fermented food samples.

Hierarchical clustering identified three distinct clusters (Fig. 4A), reflecting notable differences in microbial functional profiles at the community level among the various fermented food types. Tape ketan samples originated from black glutinous rice and white glutinous rice grouped closely together, suggesting a high degree of functional similarity. Conversely, the terasi sample was the most functionally distinct. Overall, this analysis underscores clear variations in microbial functional potential across the samples, with the terasi sample exhibiting the most unique profile.

We further quantified genes associated with beneficial metabolic functions, including the biosynthesis of bacteriocins, SCFAs (acetate, butyrate, and propionate), and vitamins B (B_1_, B_2_, B_7_, B_9_, and B_12_) and K (Fig. 4B). Bacteriocin-related genes were notably more abundant in both tape ketan samples compared to others. A distinct pattern of SCFA-related gene expression was observed across various types of fermented foods. Notably, genes involved in acetate production reached expression levels of 21 241 and 14 783 RPKM. Interestingly, the terasi sample exhibited a higher abundance of genes associated with propionate production (1 649 RPKM), while the tape singkong sample showed increased levels of genes related to butyrate synthesis (264 RPKM). The abundance of genes involved in the biosynthesis of vitamins B_1_, B_2_, B_9_, and K was relatively higher in tape ketan compared to other samples. Moreover, genes associated with vitamin B_12_ biosynthesis were more abundant in the terasi samples. Overall, although each sample exhibited distinct profiles of genes linked to beneficial metabolic functions, the tape ketan samples demonstrated the highest diversity and abundance of functional genes associated with health-promoting effects.

From a total a total of 40 MAGs were reconstructed, 26 MAGs had <10% contamination (Table S2). Among these, 18 nonredundant MAGs with a minimum of 50% completeness were successfully assembled (Fig. 4C). The predominant taxonomic classifications among these MAGs were *Lactobacillales* (*n* = 9) and *Bacillales* (*n* = 6). Nine MAGs were identified as high-quality, characterized by at least 90% completeness and <5% contamination. MAGs affiliated with *Bacillales* were predominantly observed in terasi samples, whereas MAGs associated with the order *Lactobacillales* were more commonly detected in tape ketan and tape singkong (Fig. 4C). Among the *Lactobacillales, Limosilactobacillus fermentum* exhibited the highest relative abundance, suggesting its prominent role in these fermentation environments.

Genome-centric analysis based on representative bacterial MAGs enabled detailed characterization of their potential functional contributions to health-related metabolic pathways (Fig. 4C). Several *Lactobacillales* MAGs comprising taxa such as *Enterococcus faecium, Streptococcus infantarius, Lactococcus lactis, Pediococcus pentosaceus, Leuconostoc* spp., and *Weissella* spp. harboured genes involved in acetate production, including *ack* (acetate kinase) and *pta* (phosphotransacetylase). Interestingly, these genes were also identified in MAGs affiliated with *Staphylococcales* (e.g. *Staphylococcus nepalensis* and *S. gallinarum*) and *Bacillales* (e.g. *Lentibacillus* spp.). Genes involved in the biosynthesis of vitamin B complex and vitamin K were predominantly identified in MAGs affiliated with the order *Bacillales*, particularly within the genera *Lentibacillus* and *Staphylococcus*. Notably, a MAG assigned to *S. gallinarum* contained a high number of genes associated with the biosynthetic pathways for both vitamin B complex and vitamin K. Conversely, MAGs affiliated with the order *Lactobacillales* were only primarily linked to genes involved in the biosynthesis of vitamin B2 (riboflavin) and vitamin B9 (folate). We also observed genes encoding enzymes involved in the breakdown of complex carbohydrates i.e. starch and cellulose including α-amylase and β-glucosidase, were identified across multiple MAGs. Moreover, genes encoding chitinase were also found in multiple MAGs that belong to *Bacillales* (*Bacillus* and *Lentibacillus*) and *Lactobacillales* (*Enterococcus, Lactococcus, Limosilactobacillus, Leuconostoc*, and *Weissella*). Certain genes are less frequently observed within the MAGs. For example, MAGs that were identified as *Staphylococcus, Enterococcus*, and *Leuconostoc* were found to encode β-xylosidase and pectin lyase, enzymes involved in the degradation of xylan and pectin. These results indicate a division of metabolic responsibilities among bacterial taxa in the degradation of complex carbohydrates during fermentation processes.

**Figure 5:**
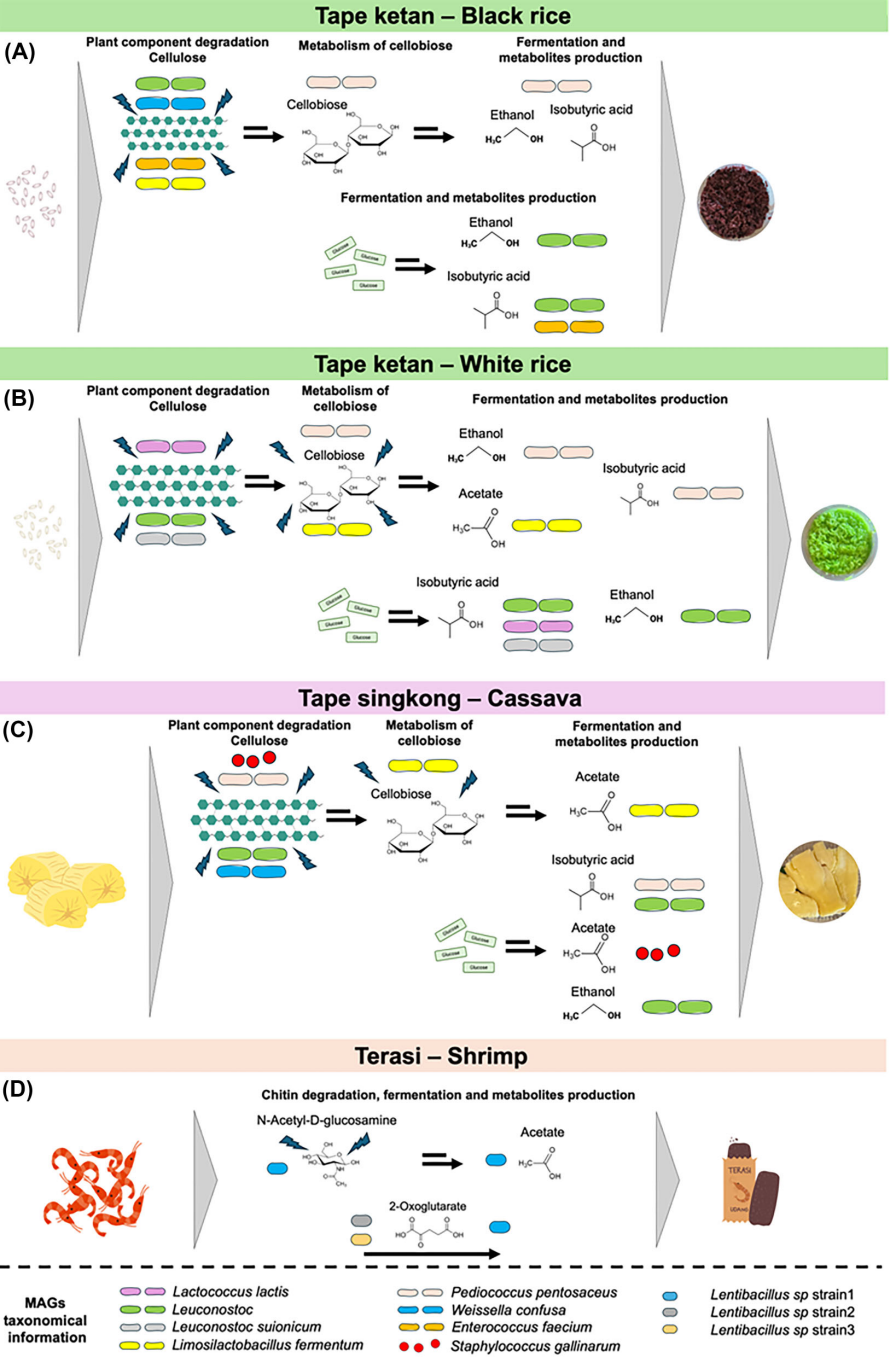
Community modelling illustrating metabolite exchanges among bacterial members in various fermented foods. Metabolic exchanges from bacterial taxa in tape ketan—black rice (A), tape ketan—white rice (B), tape singkong (C), and terasi (D) samples. Some elements were obtained from Canva (https://www.canva.com).

We conducted an in-depth analysis of metabolic interactions among bacterial taxa in traditional Indonesian fermented foods utilizing community genome-scale metabolic modelling (Fig. 5). In this analysis, we focused exclusively on bacterial interactions, as genome data is available for this group in the current study. In tape ketan produced with black rice, cellulose degradation was primarily facilitated by *L. fermentum, E. faecium, Weissella confusa*, and *Leuconostoc* species (Fig. 5A). These bacteria contributed to the production of ethanol and isobutyric acid, with *Leuconostoc* and *E. faecium* notably exporting cellobiose into the surrounding environment. The extracellular cellobiose was subsequently utilized by *P. pentosaceus*, which further contributed to ethanol and isobutyric acid synthesis. In the white rice variant of tape ketan, cellulose degradation appeared to be carried out by *L. lactis* and *Leuconostoc* species, which subsequently led to the production of ethanol and isobutyric acid (Fig. 5B). Both taxa also released cellobiose to the environment and this cellobiose was likely then metabolized by *P. pentosaceus* and *L. fermentum*, resulting in the production of ethanol, acetate, and isobutyric acid. The fermentation process of cassava in tape singkong involved a more diverse bacterial community, including *Leuconostoc, P. pentosaceus*, and *S. gallinarum*, which produced ethanol, acetate, and isobutyric acid (Fig. 5C). Additionally, cellobiose released by primary degraders was utilized by *L. fermentum*, further contributing to ethanol production. In contrast, terasi fermentation, the community metabolic modelling indicated that one *Lentibacillus* strain1 can produce acetate (Fig. 5D). These strains are likely capable of utilizing chitin as a substrate, given that their genomes include genes encoding chitinase (Fig. 4C). Metabolite exchange within the terasi microbiome was observed for 2-oxoglutarate and l-Malate. For example, *Lentibacillus* strain1 is likely to absorb 2-oxoglutarate, a metabolite released by other *Lentibacillus* strains, indicating intragenus metabolic interactions and resource sharing.

## Discussion

Our analysis reveals clear distinctions in microbial abundance, richness, and community composition among traditional Indonesian fermented foods. Tape ketan and tape singkong, both derived from starchy plant-based substrates, exhibited significantly higher microbial abundance, largely driven by the dominance of LAB. These microorganisms thrive in carbohydrate-rich environments due to their efficient fermentative metabolism of sugars (Yu et al. 2020). However, this high abundance was associated with lower microbial diversity, likely a consequence of competitive exclusion, where dominant LAB taxa, including *Lactobacillus, Leuconostoc*, and *Weissella*, suppress the growth of other microorganisms. This is further supported by the detection of abundant genes related to bacteriocin production and acetate biosynthesis in these samples, indicating antagonistic interactions that may inhibit nondominant species. In contrast, terasi, a protein- and salt-rich shrimp-based fermentation, exhibited lower microbial abundance but significantly higher bacterial richness and diversity. The limited availability of simple carbohydrates in shrimp paste likely inhibits the growth of LAB and selectively proliferates halotolerant and halophilic taxa such *Alkalibacillus, Virgibacillus, Lentibacillus, Jeotgalicoccus*, and *Tetragenococcus*. This observation aligns with previous studies of other salt-rich, marine-based fermented products such as *myeolchi-aekjeot* (a traditional Korean fish sauce) and *kapi* (a Thai fermented shrimp paste), where LAB are also found at low abundance (Lee et al. 2015, Nakamura et al. 2022).

A similar pattern in fungal community structure was observed, despite the markedly lower fungal diversity. In plant-based fermentations, a few dominant fungal taxa, such as *Rhizopus* and *Mucor*, were highly prevalent, reflecting their well-established roles as starter cultures and starch degraders in traditional Asian products (Nout and Aidoo 2010, Tamang et al. 2016). The extended fermentation period and protein- and chitin-rich nature of shrimp paste create a highly selective environment that supports the growth of halotolerant and proteolytic fungal taxa. Notably, a modest increase in the relative abundance of *Fusarium* was observed—a genus known to tolerate high salt concentrations and previously reported in the fermentation of *doenjang*, a traditional Korean salted soybean paste (Chun et al. 2020). Interestingly, the increase in richness was more pronounced than the changes observed in overall fungal diversity in terasi samples, suggesting that this environment appears to facilitate the proliferation of rare fungal species.

This study reveals a novel insight into potential cross-kingdom ecological interactions between bacterial and fungal communities. Overall, microbial communities in fermented foods are predominantly characterized by positive associations. The higher proportion of positive correlations in tape singkong, particularly between bacterial and fungal ASVs, suggests complementary metabolic interactions during fermentation. In contrast, the lower positive-to-negative ratio observed in terasi suggests a more complex and competitive microbial environment, likely due to high salinity. In tape ketan, the microbial community was predominantly composed of *Rhizopus* and specific LAB, i.e, *Pediococcus* which likely exerted selective pressure to suppress the growth of other microbial taxa. This limited diversity is reflected in the reduced complexity of the resulting microbial network. The microbial network of terasi was highly complex, dominated by interconnected hub taxa including *Staphylococcus*, a key degrader harboring chitinase genes. Similarly, we identified key fungal taxa, namely *Fusarium, Aspergillus*, and *Saccharomycopsis*, that have been previously reported to produce multiple enzymes involved in chitin degradation (Rattanakit et al. 2002, Suresh et al. 2014, Junker et al. 2019). Interestingly, despite a relatively low number of nodes, tape singkong exhibited more positive correlations between fungal and bacterial ASVs relative to other fermented products. We observed several key fungal taxa, including *Rhizopus, Mucor*, and *Saccharomycopsis*, which have been previously reported for their capacity to decompose plant fibres via the secretion of various hydrolytic enzymes (Saha 2004, Kupski et al. 2014, Farh et al. 2021). We proposed that the cassava substrate, which is high in carbohydrates and fibre and facilitates metabolic cross-feeding between starch- and fibre-degrading fungi and LAB, results in an increased cooccurrence between bacterial and fungal communities. Overall, cooccurrence network analysis further demonstrated distinct ecological network structures among the three fermented foods, which are likely influenced by differences in substrate type and fermentation processes.

Genome-centric analysis of bacterial MAGs from traditional Indonesian fermented foods revealed a diverse key metabolic function. Genes for acetate production (e.g. *ack* and *pta*) were consistently observed among LAB (*Lactobacillales*) that dominant in tape ketan and tape singkong and other *Firmicutes* (*Staphylococcales* and *Bacillales*) that are dominant in terasi. These findings indicated that functionally redundant, yet phylogenetically diverse, microbial taxa collaboratively contribute to essential metabolic processes. Genes involved in vitamin biosynthesis, particularly for vitamins B-complex and vitamin K, were predominantly associated with MAGs affiliated with *Lentibacillus* and *S. gallinarum*. In addition to *Lactobacillales, Staphylococcales*, and *Bacillales* were important taxa in plant and fish-based fermentations. Due to the production of α-amylase, β-glucosidase, and chitinase, these bacteria function as primary degraders, facilitating the breakdown of proteins into peptides and amino acids. These compounds serve as substrates for microbial metabolism and are also recognized for their role in flavour development. (Jia et al. 2021, Han et al. 2023). In addition to their role in substrate degradation, our findings also suggest that these non-LAB taxa, particularly *S. gallinarum* and *Lentibacillus* spp., may also contribute to vitamin biosynthesis, potentially enhancing the nutritional profile of the fermented products.

Community-level metabolic modelling demonstrated a cooperative and functionally integrated microbial framework that supports traditional fermented food ecosystems. In plant-based fermented foods, cellulose degradation and the production of metabolites such as ethanol and isobutyric acid were carried out by LAB. Although the dominant microbial taxa differed between rice types, the presence of extracellular cellobiose as a shared metabolite highlights its ecological role in facilitating cross-feeding interactions that shape microbial consortia. This division of metabolic labour illustrates the principle of syntrophic interactions (Zengler and Zaramela 2018), where the metabolic byproducts of one microbial group serve as substrates for another, promoting mutualistic relationships that contribute to metabolite production during fermentation. In contrast, shrimp-based terasi fermentation was predominantly driven by halophilic bacteria, particularly members of the genus *Lentibacillus*, which are well-adapted to high-salinity environments (Sundararaman et al. 2018). In this type of food, metabolite exchanged primarily involves organic acids, such as l-malate and 2-oxoglutarate. These compounds can be utilized by other members as carbon sources and for nitrogen assimilation (Meyer and Stülke 2013, Huergo and Dixon 2015), respectively.

In conclusion, our study underscores the ecological and functional complexity of microbial communities in traditional fermented foods. Functionally redundant but phylogenetically diverse taxa collaborate to support essential metabolic processes, such as substrate degradation and vitamin and SCFA biosynthesis. The findings offer a foundational framework for the targeted selection of microbial strains to improve the nutritional quality of fermented foods.

## Supplementary Material

xtag005_Supplemental_Files

## Data Availability

The sequencing data have been deposited in the European Nucleotide Archive (ENA) under project number PRJEB100938.
