# Supplementary material for: Traditional fermented foods of Indonesia harbour functionally redundant but phylogenetically diverse taxa
Source: FEMS Microbes. 2026 Jan 22;7:xtag005. doi: 10.1093/femsmc/xtag005 (PMC12875122; doi:10.1093/femsmc/xtag005)
Supplement: xtag005_Supplemental_Files [file xtag005_supplemental_files.zip › FEMSMC-2025-055.R1 one sentence summary.docx]

This study investigates the microbial and functional diversity of traditional fermented foods in Indonesia. Plant-based fermented products demonstrated higher bacterial abundance, but lower diversity compared to animal-based terasi.
