# Supplementary material for: Traditional fermented foods of Indonesia harbour functionally redundant but phylogenetically diverse taxa
Source: FEMS Microbes. 2026 Jan 22;7:xtag005. doi: 10.1093/femsmc/xtag005 (PMC12875122; doi:10.1093/femsmc/xtag005)
Supplement: xtag005_Supplemental_Files [file xtag005_supplemental_files.zip › Supplementary Materials.docx]

**Traditional fermented foods of Indonesia harbor functionally redundant but phylogenetically diverse taxa**

**Wisnu Wicaksono^1,^ Elma Zukancic^1^, Matevz Zlatnar^1^, Antonius Suwanto^2^, Gabriele Berg^1,3,4,5#^,**

^1^Institute of Environmental Biotechnology, Graz University of Technology, Graz, Austria

^2^Department of Biology, Faculty of Mathematics and Natural Science, IPB University, Bogor, Indonesia

^3^Leibniz Institute for Agricultural Engineering and Bioeconomy (ATB), Potsdam, Germany

^4^Institute for Biochemistry and Biology, University of Potsdam, Potsdam, Germany

^5^Department of Colloid Chemistry, Max Planck Institute of Colloids and Interfaces, Potsdam, Germany

^#^Corresponding authors:

Wisnu Adi Wicaksono and Gabriele Berg, Graz University of Technology, Graz

Emails: [wisnu.wicaksono@tugraz.at](mailto:wisnu.wicaksono@tugraz.at) & gabriele.berg@tugraz.at


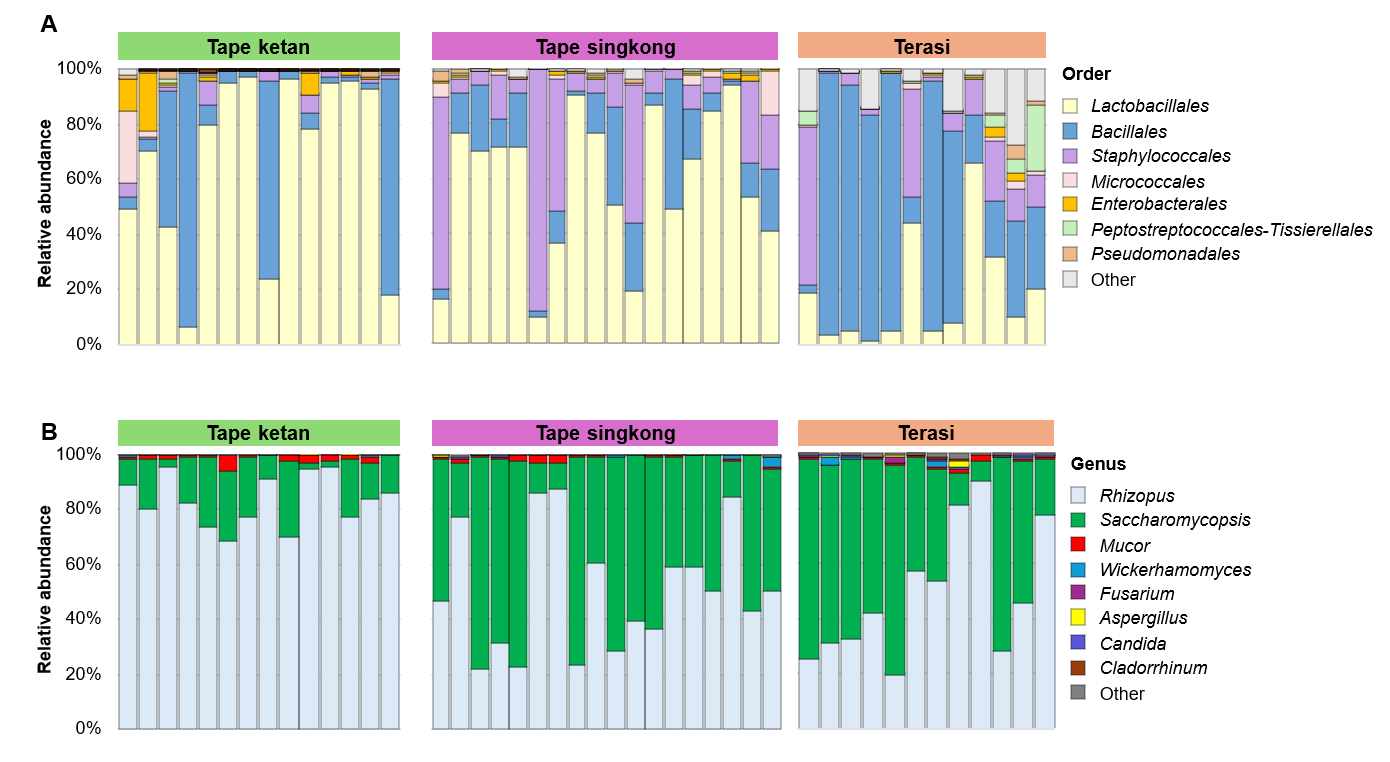


**Supplementary Figure S1 Microbial community composition in various fermented foods**. Bar plots show bacterial (A) and fungal (B) composition at order and genus levels for different fermented food samples, respectively.

**Supplementary Table S1 Detail of sampling location of fermented food products**

| Sample ID | Fermented food product | Fermented food type | Province |
| --- | --- | --- | --- |
| TK1 | Tape ketan | Plant | Bali |
| TK2 | Tape ketan | Plant | East Java |
| TK3 | Tape ketan | Plant | East Java |
| TK4 | Tape ketan | Plant | West Java |
| TK5 | Tape ketan | Plant | West Java |
| TK6 | Tape ketan | Plant | Bali |
| TK7 | Tape ketan | Plant | Bali |
| TK8 | Tape ketan | Plant | Bali |
| TK9 | Tape ketan | Plant | Bali |
| TK10 | Tape ketan | Plant | Bali |
| TK11 | Tape ketan | Plant | East Java |
| TK12 | Tape ketan | Plant | East Java |
| TK13 | Tape ketan | Plant | DKI Jakarta |
| TK14 | Tape ketan | Plant | DKI Jakarta |
| TR2 | Terasi | Animal | Central Java |
| TR3 | Terasi | Animal | East Java |
| TR4 | Terasi | Animal | East Java |
| TR5 | Terasi | Animal | East Java |
| TR6 | Terasi | Animal | East Java |
| TR7 | Terasi | Animal | East Java |
| TR8 | Terasi | Animal | East Java |
| TR9 | Terasi | Animal | Bali |
| TR10 | Terasi | Animal | East Java |
| TR11 | Terasi | Animal | Bali |
| TR12 | Terasi | Animal | Bali |
| TR13 | Terasi | Animal | East Java |
| TS1 | Tape singkong | Plant | Bali |
| TS2 | Tape singkong | Plant | East Java |
| TS3 | Tape singkong | Plant | East Java |
| TS4 | Tape singkong | Plant | Bali |
| TS5 | Tape singkong | Plant | East Java |
| TS6 | Tape singkong | Plant | East Java |
| TS7 | Tape singkong | Plant | Bali |
| TS8 | Tape singkong | Plant | Bali |
| TS9 | Tape singkong | Plant | East Java |
| TS10 | Tape singkong | Plant | Bali |
| TS11 | Tape singkong | Plant | Bali |
| TS12 | Tape singkong | Plant | Bali |
| TS13 | Tape singkong | Plant | East Java |
| TS14 | Tape singkong | Plant | DKI Jakarta |
| TS15 | Tape singkong | Plant | DKI Jakarta |
| TS16 | Tape singkong | Plant | DKI Jakarta |
| TS17 | Tape singkong | Plant | DKI Jakarta |
| TS18 | Tape singkong | Plant | West Java |

**Supplementary Table S2 Detailed assembly features and taxonomic classification of bacterial MAGs from various fermented foods.** MAGs that are highlighted in bold and coloured red are non-redundant AMGs.

| GenomeID | Compl. (%) | Contam (%) | Taxonomical information |
| --- | --- | --- | --- |
| MAG_TK3_1 | 96.18 | 1.87 | *Bacteria;Firmicutes;Bacilli;Lactobacillales;Streptococcaceae;Streptococcus;Streptococcus infantarius* |
| **MAG_TK3_2** | 98.09 | 1.09 | *Bacteria;Firmicutes;Bacilli;Lactobacillales;Lactobacillaceae;Limosilactobacillus;Limosilactobacillus fermentum* |
| **MAG_TK3_3** | 98.87 | 6.4 | *Bacteria;Firmicutes;Bacilli;Lactobacillales;Enterococcaceae;Enterococcus;Enterococcus faecium* |
| MAG_TK3_4 | 13.36 | 3.98 | *Bacteria;Firmicutes;Bacilli;Lactobacillales;Lactobacillaceae;Pediococcus;* |
| **MAG_TK3_5** | 78.62 | 1.72 | *Bacteria;Firmicutes;Bacilli;Lactobacillales;Lactobacillaceae;Pediococcus;Pediococcus pentosaceus* |
| **MAG_TK3_6** | 83.37 | 0.89 | *Bacteria;Firmicutes;Bacilli;Lactobacillales;Lactobacillaceae;Weissella;Weissella paramesenteroides* |
| MAG_TK3_7 | 58.52 | 32.62 | *Bacteria;Firmicutes;Bacilli;Lactobacillales;Lactobacillaceae;Pediococcus;* |
| MAG_TK3_8 | 87.72 | 35.12 | *Bacteria;Firmicutes;Bacilli;Lactobacillales;Lactobacillaceae;Weissella;Weissella confusa* |
| **MAG_TK5_1** | 97.13 | 5.63 | *Bacteria;Firmicutes;Bacilli;Lactobacillales;Streptococcaceae;Lactococcus;Lactococcus lactis* |
| **MAG_TK5_2** | 99.37 | 1.26 | *Bacteria;Firmicutes;Bacilli;Lactobacillales;Lactobacillaceae;Leuconostoc;* |
| **MAG_TK5_3** | 91.96 | 0.88 | *Bacteria;Firmicutes;Bacilli;Lactobacillales;Lactobacillaceae;Leuconostoc;Leuconostoc suionicum* |
| MAG_TK5_4 | 98.63 | 4.1 | *Bacteria;Firmicutes;Bacilli;Lactobacillales;Lactobacillaceae;Limosilactobacillus;Limosilactobacillus fermentum* |
| MAG_TK5_5 | 33.39 | 5.86 | *Bacteria;Proteobacteria;Gammaproteobacteria;Enterobacterales;Aeromonadaceae;Tolumonas;* |
| MAG_TK5_6 | 56.79 | 17.01 | *Archaea;Asgardarchaeota;Heimdallarchaeia;;;;* |
| MAG_TK5_7 | 59.87 | 34.33 | *Archaea;Asgardarchaeota;Heimdallarchaeia;;;;* |
| MAG_TK5_8 | 81.39 | 39.3 | *Bacteria;Proteobacteria;Gammaproteobacteria;Pseudomonadales;Moraxellaceae;Acinetobacter;* |
| MAG_TK5_9 | 70.45 | 46.93 | *Bacteria;Proteobacteria;Gammaproteobacteria;Enterobacterales;Enterobacteriaceae;Phytobacter;* |
| MAG_TK5_10 | 96.98 | 78.71 | *Bacteria;Firmicutes;Bacilli;Lactobacillales;Lactobacillaceae;Lactiplantibacillus;* |
| **MAG_TS5_1** | 50.21 | 3.78 | *Bacteria;Firmicutes;Bacilli;Bacillales;Bacillaceae;Bacillus;Bacillus megaterium* |
| MAG_TS5_2 | 31.05 | 0.41 | *Bacteria;Firmicutes;Bacilli;Lactobacillales;Lactobacillaceae;Limosilactobacillus;Limosilactobacillus fermentum* |
| MAG_TS5_3 | 11.61 | 0.05 | *Bacteria;Proteobacteria;Gammaproteobacteria;Enterobacterales;Enterobacteriaceae;Enterobacter;Enterobacter roggenkampii* |
| MAG_TS5_4 | 35.09 | 3.37 | *Bacteria;Firmicutes;Bacilli;Bacillales;Bacillaceae;Bacillus;* |
| **MAG_TS5_5** | 99.45 | 0.55 | *Bacteria;Firmicutes;Bacilli;Staphylococcales;Staphylococcaceae;Staphylococcus;Staphylococcus gallinarum* |
| **MAG_TS5_6** | 95.51 | 1.31 | *Bacteria;Firmicutes;Bacilli;Lactobacillales;Streptococcaceae;Streptococcus;Streptococcus infantarius* |
| **MAG_TS5_7** | 90.08 | 0 | *Bacteria;Firmicutes;Bacilli;Lactobacillales;Lactobacillaceae;Weissella;Weissella confusa* |
| MAG_TS5_8 | 59.23 | 10.43 | *Bacteria;Firmicutes;Bacilli;Lactobacillales;Enterococcaceae;Enterococcus;Enterococcu italicus* |
| MAG_TS5_9 | 30.31 | 10.63 | *Archaea;Asgardarchaeota;Heimdallarchaeia;;;;* |
| MAG_TS5_10 | 96.77 | 15.67 | *Bacteria;Firmicutes;Bacilli;Lactobacillales;Lactobacillaceae;Leuconostoc;Leuconostoc sp900554745* |
| MAG_TS5_11 | 58.31 | 30.66 | *Archaea;Thermoproteota;Nitrososphaeria;QMWL01;;;* |
| MAG_TS5_12 | 99.81 | 97.61 | *Bacteria;Firmicutes;Bacilli;Bacillales;Bacillaceae;Bacillus;Bacillus velezensis* |
| **MAG_TR3_1** | 64.45 | 2.44 | *Bacteria;Firmicutes;Bacilli;Bacillales;Halobacillaceae;Salimicrobium;Salimicrobium album* |
| **MAG_TR3_2** | 98.28 | 7.77 | *Bacteria;Firmicutes;Bacilli;Bacillales;Amphibacillaceae;Lentibacillus;* |
| **MAG_TR3_3** | 93.19 | 2.69 | *Bacteria;Firmicutes;Bacilli;Staphylococcales;Staphylococcaceae;Staphylococcus;Staphylococcus nepalensis* |
| **MAG_TR3_4** | 94.7 | 0.02 | *Bacteria;Firmicutes;Bacilli;Bacillales;Amphibacillaceae;Lentibacillus;* |
| **MAG_TR3_5** | 76.6 | 2.06 | *Archaea;Halobacteriota;Halobacteria;Halobacteriales;Halococcaceae;Halococcus;Halococcus thailandensis* |
| **MAG_TR3_6** | 89.36 | 0 | *Bacteria;Firmicutes;Bacilli;Bacillales;Amphibacillaceae;Lentibacillus;* |
| **MAG_TR3_7** | 93.73 | 0.66 | *Bacteria;Firmicutes;Bacilli;Bacillales;Amphibacillaceae;Lentibacillus;* |
| MAG_TR3_8 | 18.01 | 0 | *Bacteria;Firmicutes;Bacilli;Bacillales;Amphibacillaceae;Lentibacillus;* |
| MAG_TR3_9 | 52.82 | 12.19 | *Bacteria;Firmicutes;Bacilli;Staphylococcales;Salinicoccaceae;Salinicoccus;Salinicoccus roseus* |
| MAG_TR3_10 | 82.13 | 18.31 | *Bacteria;Bacteroidota;Bacteroidia;Chitinophagales;Chitinophagaceae;Sediminibacterium;* |
